# Bck2 Acts through the MADS Box Protein Mcm1 to Activate Cell-Cycle-Regulated Genes in Budding Yeast

**DOI:** 10.1371/journal.pgen.1003507

**Published:** 2013-05-09

**Authors:** Nazareth Bastajian, Helena Friesen, Brenda J. Andrews

**Affiliations:** The Donnelly Centre and the Department of Molecular Genetics, University of Toronto, Toronto, Ontario, Canada; Fred Hutchinson Cancer Research Center, United States of America

## Abstract

The Bck2 protein is a potent genetic regulator of cell-cycle-dependent gene expression in budding yeast. To date, most experiments have focused on assessing a potential role for Bck2 in activation of the G_1_/S-specific transcription factors SBF (Swi4, Swi6) and MBF (Mbp1, Swi6), yet the mechanism of gene activation by Bck2 has remained obscure. We performed a yeast two-hybrid screen using a truncated version of Bck2 and discovered six novel Bck2-binding partners including Mcm1, an essential protein that binds to and activates M/G_1_ promoters through Early Cell cycle Box (ECB) elements as well as to G_2_/M promoters. At M/G_1_ promoters Mcm1 is inhibited by association with two repressors, Yox1 or Yhp1, and gene activation ensues once repression is relieved by an unknown activating signal. Here, we show that Bck2 interacts physically with Mcm1 to activate genes during G_1_ phase. We used chromatin immunoprecipitation (ChIP) experiments to show that Bck2 localizes to the promoters of M/G_1_-specific genes, in a manner dependent on functional ECB elements, as well as to the promoters of G_1_/S and G_2_/M genes. The Bck2-Mcm1 interaction requires valine 69 on Mcm1, a residue known to be required for interaction with Yox1. Overexpression of *BCK2* decreases Yox1 localization to the early G_1_-specific *CLN3* promoter and rescues the lethality caused by overexpression of *YOX1*. Our data suggest that Yox1 and Bck2 may compete for access to the Mcm1-ECB scaffold to ensure appropriate activation of the initial suite of genes required for cell cycle commitment.

## Author Summary

Cell-cycle-dependent gene expression is a universal feature of cell cycles, with clear transcriptional programs in yeast, bacteria, and metazoans. At the M/G_1_ transition, many of the up-regulated genes encode key regulators of DNA replication (*CDC6*) and cyclins that initiate the events of cell cycle commitment (*PCL9*, *CLN3*). The promoters of genes activated at M/G_1_ contain a cis-regulatory sequence called the early cell cycle box (ECB), which is bound by the MADS-box transcription factor Mcm1, as well as the repressor Yox1 or Yhp1. The ECB cluster of genes defines a crucial cell cycle window during which a cell may change its fate; yet how the regulators that appear to act at ECBs are linked to cell cycle position is unclear, and coregulators, which experience tells us must exist, were unknown. Here, we describe our discovery that Bck2, a potent cell-cycle-regulator whose function has remained obscure, functions as a cofactor for Mcm1, to induce ECB–dependent gene expression. We also show that Bck2 has a role in promoting expression of late G_1_ and M/G_2_ genes. Our genetic and biochemical experiments reveal a new pathway for regulating gene expression associated with early cell cycle commitment, a process that is highly conserved.

## Introduction

The temporal control of transcription is likely a universal feature of cell cycles, with clear transcriptional programs in yeast, bacteria and metazoans [1]–[4]. Bursts of gene expression in eukaryotes tend to be associated with major cell cycle transitions that are governed by cyclin-dependent kinases (Cdks) in association with regulatory subunits called cyclins [5]. Up-regulation of G_1_-specific forms of the Cdc28 Cdk is rate-limiting for cell cycle commitment. Control over Cdc28 activity is exerted at several levels, including transcriptional induction of cyclin gene expression; the G_1_/S phase transition features activation of a massive transcriptional program of ∼120 genes, including the G_1_ cyclin genes *CLN1* and *CLN2* [6]–[10]. Two heterodimeric transcription factors largely drive the G_1_/S cluster: SBF (Swi4,6-dependent cell cycle box binding factor, a heterodimer of Swi4 and Swi6) binds the so-called SCB promoter element (Swi4,6-dependent cell cycle box) found upstream of the cyclin genes and cell wall biosynthetic genes, while MBF (*Mlu*I cell cycle box binding factor, a heterodimer of Mbp1 and Swi6) preferentially acts through a distinct element called the MCB (*Mlu*I cell cycle box) found mostly upstream of DNA replication and repair genes.

Although the role of SBF and MBF at the G_1_/S transition is well established, deletion of Swi6, the common subunit of both complexes, does not cause cell cycle arrest, suggesting alternative pathways for activating G_1_ transcription. One of these pathways is defined by Bck2, a poorly understood cell cycle regulator whose deletion causes dramatic cell cycle phenotypes in certain genetic contexts. For example, deletion of both *CLN3*, which encodes a cyclin that activates Cdc28 at M/G_1_, and *BCK2* causes synthetic lethality [11]–[13]. Furthermore, *BCK2* is one of only two genes whose overexpression is known to bypass the lethality caused by mutation of all three G_1_-specific Cdc28 cyclins (*CLN1*, *CLN2* and *CLN3*) [11]. Bck2 was discovered almost 20 years ago in screens for high copy suppressors of protein kinase C pathway mutants (including *pkc1* itself) and G_1_ cyclin deficiencies [11], [14] and is thought to have an activating role in transcription of G_1_/S genes [12], [13]. Activation of G_1_/S genes by Bck2 depends partly, but not wholly, on SBF and MBF and Bck2 appears capable of activating G_1_/S transcription in the absence of Cdc28, indicating SBF- and MBF-independence [13]. Furthermore, a region in the promoter of *CLN2*, termed UAS2, which completely lacks SCB or MCB elements, is activated by *BCK2* overexpression [12] and operates in a *CDC28*-independent manner [15]. Bck2 also has a role in regulating heat shock genes and adaptation of the protein kinase C pathway MAP kinase Slt2 [16], although it is not clear whether these roles depend on SBF. Collectively, these findings suggest that Bck2 may operate through an unidentified DNA-binding factor whose activity is Cdc28-independent.

The MADS box transcription factor Mcm1 has an important regulatory function at two points in the cell cycle – M/G_1_ and G_2_/M. During M/G_1_, Mcm1 functions as a critical constituent of a complex that forms on ECB (Early Cell cycle Box) elements in promoters of genes expressed at the M/G_1_ phase transition such as *CLN3* and *SWI4* [17]. Two related homeodomain proteins, Yox1 and Yhp1, act as repressors of Mcm1 on ECB elements by physically interacting with Mcm1 and with DNA binding sites next to the Mcm1 site in the ECB element [18]. Mcm1 is also a critical constituent of complexes that form during the G_2_/M phase transition to control the *CLB2* cluster of genes, including the G_2_ cyclin gene *CLB2* and *CDC20*, which encodes an activator of the anaphase promoting complex [19]. The *CLB2* gene cluster is activated by the Clb-Cdc28 and Cdc5 kinases [20]–[23] and negatively regulated by protein kinase C, Pkc1 [24]; these kinases regulate a promoter-bound complex of Mcm1, Fkh2 and Ndd1 [25]–[29]. Some *CLB2* cluster genes, but not *CLB2* itself, contain hybrid elements composed of an Mcm1-binding site flanked by Yox1- and Fkh2-binding sites [18]. In *CLB2* cluster genes that contain such elements, Yox1 and Fkh2 compete for binding to DNA-bound Mcm1 despite the spatial separation of their DNA recognition elements [30]. Interestingly, no such juxtaposed binding motifs are obvious in the vicinity of ECB elements and no other binding partners for Mcm1 that positively regulate these genes have been identified. Mcm1 also has roles at the promoters of some other genes, including the G_1_/S gene *CLN2*, where it contributes to the presence of a nucleosome depleted region that is needed for reliable “on/off” expression once per cell cycle [31].

In this study, we show that Bck2 activates expression of the M/G_1_ genes *CLN3* and *SWI4*, through an interaction with Mcm1 on ECB elements in the promoters of these genes. Moreover, increased Bck2 dosage leads to decreased Yox1 binding at M/G_1_ promoters and overproduction of *BCK2* rescues the lethality caused by overexpression of *YOX1*, indicating that Bck2 and Yox1 may compete for access to Mcm1 on promoters. Consistent with this hypothesis, mutation of a key residue on Mcm1 known to prevent interaction with Yox1 also prevents interaction with Bck2. In addition, we show that Bck2 interacts with the promoters of the G_1_/S gene *CLN2* and the G_2_/M gene *CLB2*. Our experiments reveal a previously unappreciated function for Bck2 as a cofactor for Mcm1 and suggest a more general role for Bck2 in regulating cell-cycle gene expression.

## Results

### Truncation Analysis of the *BCK2* Gene

**Figure 1:**
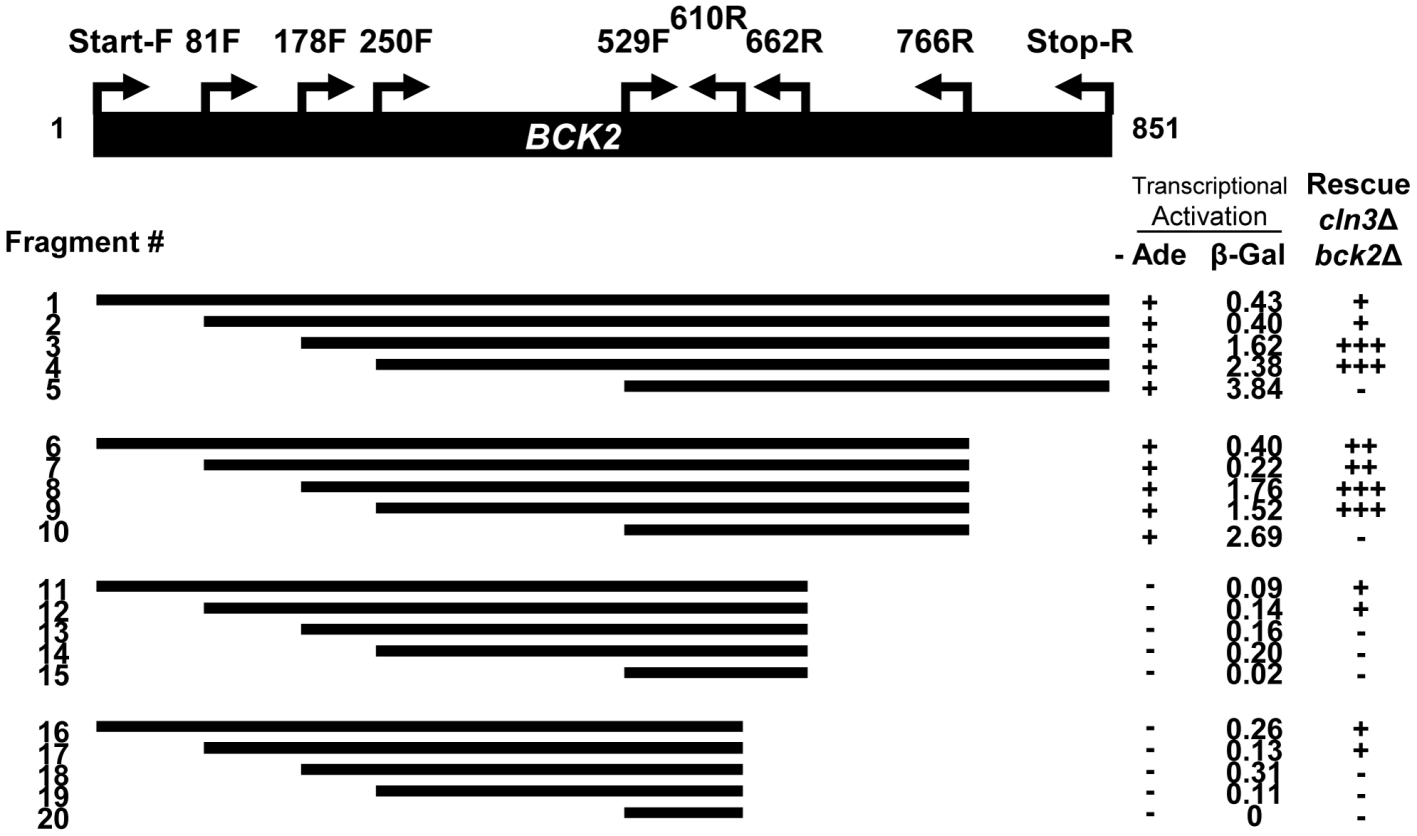
Truncation analysis of the *BCK2* gene. Fragments of the *BCK2* gene (black bar) were amplified from genomic DNA using primer positions shown and designated as “amino acid+F (Forward), or R (reverse)”. PCR products were cloned into a yeast two-hybrid vector to create *BCK2* fragments fused to the N-terminal *GAL4* DBD (DNA Binding Domain). High density growth spots (in either the *ADE2* transcription activation assay or the complementation assay) were called “+”, “++”, or “+++” depending on extent of growth. A complete absence of growth was called “−”. Numbers in the β-Gal column represent averaged quantities (per fusion protein) in Miller Units (U).

We reasoned that an exploration of Bck2-interacting proteins might illuminate the function of Bck2 in G_1_ progression. Bck2 is difficult to work with biochemically (for example endogenous Bck2 is undetectable by Western blot [16], [32, and data not shown], so we turned to the yeast two-hybrid (Y2H) system for our protein interaction screens. Bck2 has a potent transcriptional activation domain, and activates reporter gene expression when fused to the Gal4 DNA binding domain (DBD) in a Y2H reporter strain [33] (Figure 1), a property that has precluded identification of Bck2 binding partners using the Y2H screening method. To discover Bck2 derivatives that might be useful for two-hybrid screening, we created 20 truncations of the *BCK2* gene and fused them to the *GAL4 DBD* (Figure 1). We first assessed the ability of each truncation construct to activate transcription of two reporter genes, in which the *GAL4* UAS is upstream of either *lacZ* (Figure S1) or *ADE2* (Figure S1B). Bck2 residues 662 to 851 were required for transcriptional activation (fragments 11–20), while the Bck2 N-terminal region had some apparent inhibitory activity (Figure S1A), as suggested by our finding that deletions of the N-terminus resulted in significant increases in reporter gene expression. A construct containing fragment 5, lacking the first 529 amino acids of Bck2, was the most potent Y2H auto-activator in the *lacZ* reporter assay (Figure S1A).

Proteins that autoactivate in the Y2H system often have a role in transcription and this property may reflect the biological activity of the protein [33]. To explore the relationship between Y2H autoactivation and biological function, we assessed the ability of each Bck2 fragment to complement the synthetic lethal phenotype of a *cln3*Δ*bck2*Δ strain (Figure S1C). We discovered that a fragment of Bck2 containing residues 250 to 766 was able to robustly complement the lethality of the *cln3*Δ*bck2*Δ strain (Figure 1, Figure S1C). Consistent with the observation that the first 178 residues of Bck2 are not necessary for suppression of the *pkc1* lysis phenotype by high-copy *BCK2* [14], a derivative of Bck2 lacking the N-terminal 178 residues was also able to complement the inviability of the *cln3*Δ*bck2*Δ strain (Figure S1C). Bck2 residues 529 to 851 alone failed to complement the *cln3*Δ*bck2*Δ phenotype but were sufficient for Y2H auto-activation. This region is also insufficient to suppress the lysis phenotype of *pkc1* mutants [14]. We conclude that the central region (250 to 766) of Bck2 lacking the N- and C-terminal ends is sufficient to complement essential *in vivo* functions of Bck2 and that this essential function is separable from the Y2H auto-activation activity of the Bck2 protein.

### A Yeast-Two-Hybrid Screen Using Gal4 DBD-Bck2 as Bait Identifies Six Novel Interacting Proteins

Thus far, no known protein interaction partners of Bck2 easily explain the cell cycle transcription phenotypes associated with deletion of *BCK2*. To carry out a Y2H screen, we decided to use the largest Bck2 construct that did not auto-activate the *ADE2* Y2H reporter gene, but complemented the inviability of the *cln3*Δ*bck2*Δ strain (fragment 11; Figure 1). We chose this fragment for our screen since complementing regions often contain important protein-protein interaction domains. For example, the minimal region of the Ada2 protein required for complementation is the same region required for physical interaction with Gcn5 and Ada3 [34].

**Figure 2:**
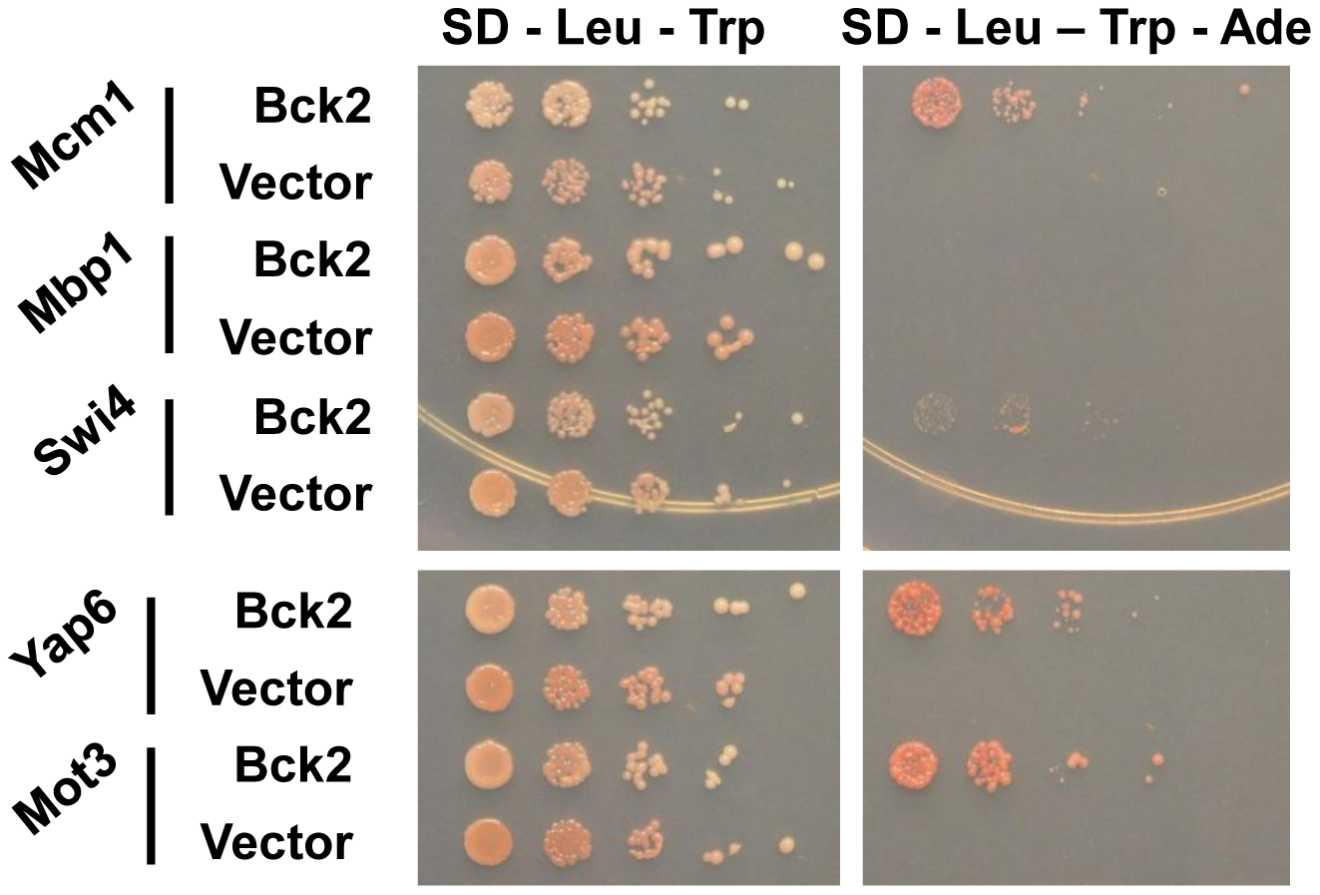
Bck2-interacting proteins identified in a genome-wide yeast two-hybrid screen. Yeast transformants carrying *ADH1*-*GAL4* DBD (vector; *LEU2*) or *ADH1*-*GAL4* DBD-*BCK2* Fragment 11 (Bck2) in a two-hybrid bait strain (Y8930) were mated to yeast transformants of a two-hybrid prey strain (Y8800) bearing specific gene ORFs fused to the N-terminal *GAL4* AD (activation domain; *TRP1*, i.e. *ADH1*-*GAL4* AD-ORF plasmid). Diploids were selected by streaking on double plasmid selection medium (SD – Leu – Trp). Strains were grown to equivalent optical density, and spotted in serial 10-fold dilutions on double plasmid selection medium (SD – Leu – Trp) or medium where growth is proportional to transcription of the *ADE2* gene (SD – Leu – Trp - Ade). Plates were incubated for 48 h at 30°C.

We used the ORFeome Y2H screening method [35] to discover potential Bck2-interacting proteins. We identified six proteins that interacted with Bck2: Mcm1, Yap6, Tpd3, Std1, Mth1, and Mot3 (Figure 2 and data not shown). With the exception of Tpd3, all of these proteins are transcriptional regulators that act proximal to DNA. As noted in the introduction, Mcm1 is a member of a class of MADS box transcription factors found in all eukaryotic organisms [36]–[38] and we explore the Mcm1-Bck2 interaction in detail below. Four of the other Bck2 partners we identified in our screen were also DNA binding proteins that had roles in ion homeostasis and nutrient sensing: (1) Yap6 is a basic leucine zipper (bZIP) transcription factor that activates a number of genes involved in sodium and lithium tolerance [39], [40]; (2) Std1 and Mth1 are both controllers of glucose-regulated gene expression [41] and are required for transcriptional repression of *HXT* (hexose transport) genes [42], [43]; (3) Mot3 is a Zn-finger transcription factor that activates a number of cell wall genes [44], and represses transcription of the *DAN*/*TIR* group of genes that encode cell wall mannoproteins during anaerobic growth [44]. The only protein that is not a transcription factor, Tpd3, is the scaffold subunit A of the heterotrimeric protein phosphatase 2A (PP2A) [45], which has roles at several points in the cell cycle and is involved in the *TOR* pathway for nutrient sensing [46]. We did not identify *SWI4* or *MBP1* in our Y2H screen, but a direct test revealed a weak interaction between Bck2 and Swi4 (Figure 2). The identification of genes with known roles in transcription and nutrient response is consistent with the apparent functions of Bck2, and validates our two-hybrid screen as a tool for identifying Bck2-interacting proteins.

### Bck2 Activates Cell-Cycle-Regulated Genes that Contain Mcm1-Binding Sites in Their Promoters

**Figure 3:**
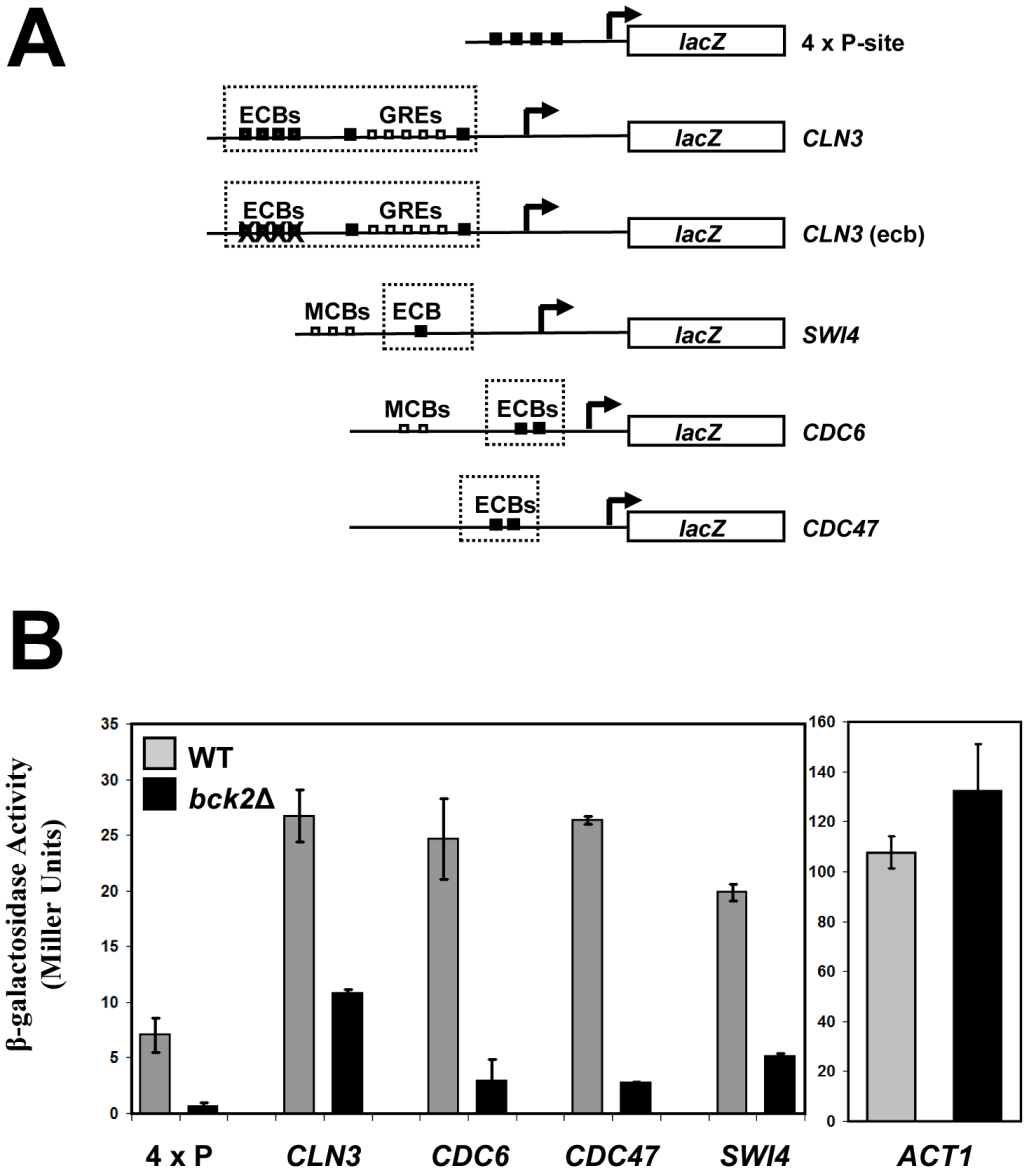
Bck2 activates Mcm1-driven *lacZ* reporter constructs. (A) Diagrams of plasmid reporter constructs used to assess the effect of *BCK2* deletion or overexpression. Constructs containing either multiple synthetic Mcm1-binding sites upstream of the *lacZ* gene (4 x P-site, pCLM771) or endogenous promoters that contain ECB elements (*CLN3* pBD1790, *SWI4* pBD1577, *CDC6* pBD1637, *CDC47* pBD1951) are shown. Black boxes represent distinct Mcm1-binding sites such as Mcm1-binding P-site elements or ECB elements, whereas white boxes represent MCB elements or GRE elements. (B) WT (grey bars) or *bck2*Δ (black bars) yeast transformants carrying P-*lacZ*, *CLN3*-*lacZ*, *SWI4*-*lacZ*, *CDC6*-*lacZ*, *CDC47*-*lacZ*, and *ACT1*-*lacZ* were assessed for *lacZ* expression level. Asynchronous cells were grown to mid-log phase in selective medium and subjected to quantitative β-galactosidase assays to measure *lacZ* expression. Y-axis values are expressed in Miller units. Error bars reflect values obtained from 3 independent transformants in separate experiments.

**Figure 4:**
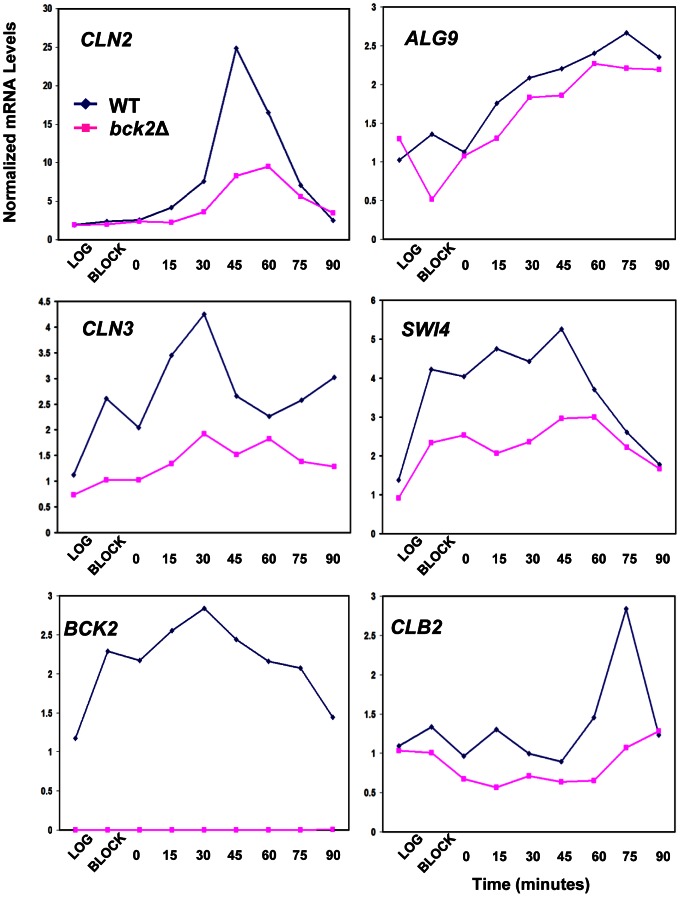
Effect of *BCK2* deletion on *CLN2*, *ALG9*, *CLN3*, *SWI4*, *BCK2*, and *CLB2* mRNA accumulation during the cell cycle. Y8890 (*cdc20*-*3* WT, dark blue line with diamonds) and BY4897 cultures (*cdc20*-*3 bck2*Δ, dark pink line with squares) were grown to log-phase, arrested at M/G_1_ by incubating for 3.5 hours at 37°C (block), then released into the cell cycle by re-incubation at 21°C. Samples were harvested every 15 minutes and mRNA levels quantified by Q-PCR using *ACT1* mRNA levels as a normalizing control. In WT cells, the peak of *CLN2* transcription marks the G_1_/S transition, the peak of *CLN3* and *SWI4* marks M/G_1_, and the peak of *CLB2* marks G_2_/M.

Given the clear roles for Mcm1 in cell-cycle-dependent gene expression, we chose to focus our follow-up analysis on the Mcm1-Bck2 interaction. In addition to the Y2H interaction between Bck2 and Mcm1, several observations from earlier studies implicate Bck2 in the activation of Mcm1 target genes in M/G_1_ phase: (1) mRNA from the M/G_1_ gene *SWI4* accumulates much more slowly in *bck2*Δ than WT cells in synchronized cultures, whereas *SWI4* is upregulated in cells that overexpress *BCK2* [12]; (2) high-copy *BCK2* stimulates the expression of the Mcm1-dependent reporter gene, P-*lacZ* [47]; (3) overexpression of *BCK2* causes increased transcription of *CLN3* and *SWI4* by microarray analysis [48]. However, up to now, no direct connection between *BCK2* and M/G_1_ genes has been established. To evaluate the significance of the Mcm1-Bck2 physical interaction *in vivo*, we first assessed the effect of *BCK2* deletion on expression of *lacZ* reporter genes whose expression was dependent on either multiple Mcm1-binding sites (4 x P-sites) [47] or the upstream activating sequences of four Mcm1-regulated genes expressed in M/G_1_ phase – *CLN3*, *CDC6*, *CDC47*, *SWI4* [49] (Figure 3A). In these plasmid reporter assays, deletion of *BCK2* had no effect on expression of a control *ACT1*-*lacZ* reporter gene. However, we saw a pronounced reduction in expression of the *CLN3*-*lacZ*, *CDC6*-*lacZ*, *CDC47*-*lacZ*, *SWI4*-*lacZ*, and P-*lacZ* reporter genes in the *bck2*Δ strain (Figure 3B). These results suggest a role for *BCK2* in M/G_1_ gene expression. To verify the results of the reporter gene assays, we next examined endogenous levels of Mcm1 target gene expression in a *bck2*Δ strain (Figure 4). Since Mcm1 controls *CLN3* and *SWI4* transcript accumulation at a very early point in G_1_, we synchronized cultures in mitosis with a *cdc20*-*3* temperature-sensitive allele and released them into the subsequent cell cycle. Cells in this experiment were slow growing and enriched in large-budded cells that precluded FACS analysis of cell cycle synchrony (data not shown). However, *CLN2* transcription was highly periodic in both WT and *bck2*Δ cells, indicating that these cultures were synchronously released from the mitotic block. Consistent with previous reports, *CLN2* transcript was reduced in the *bck2*Δ strain [11], [12] while levels of a control transcript (*ALG9*) were unaffected. Strikingly, the accumulation of *CLN3* and *SWI4* mRNAs was significantly reduced in *bck2*Δ cells, and peak expression was also delayed, at least for the *CLN3* transcript. In wild-type cells, *CLB2* mRNA peaked after G_1_ transcripts as expected. However, in *bck2*Δ cells *CLB2* transcripts were delayed and only began to accumulate near the end of the time-course, after the peak of *CLB2* expression seen in wild-type cells. Thus, *BCK2* is required for the appropriate expression of the M/G_1_ genes *CLN3* and *SWI4*, the G_1_/S gene *CLN2*, and the G_2_/M gene *CLB2*.

### Bck2 Requires ECB Elements for Transcriptional Activation of M/G_1_ Genes

**Figure 5:**
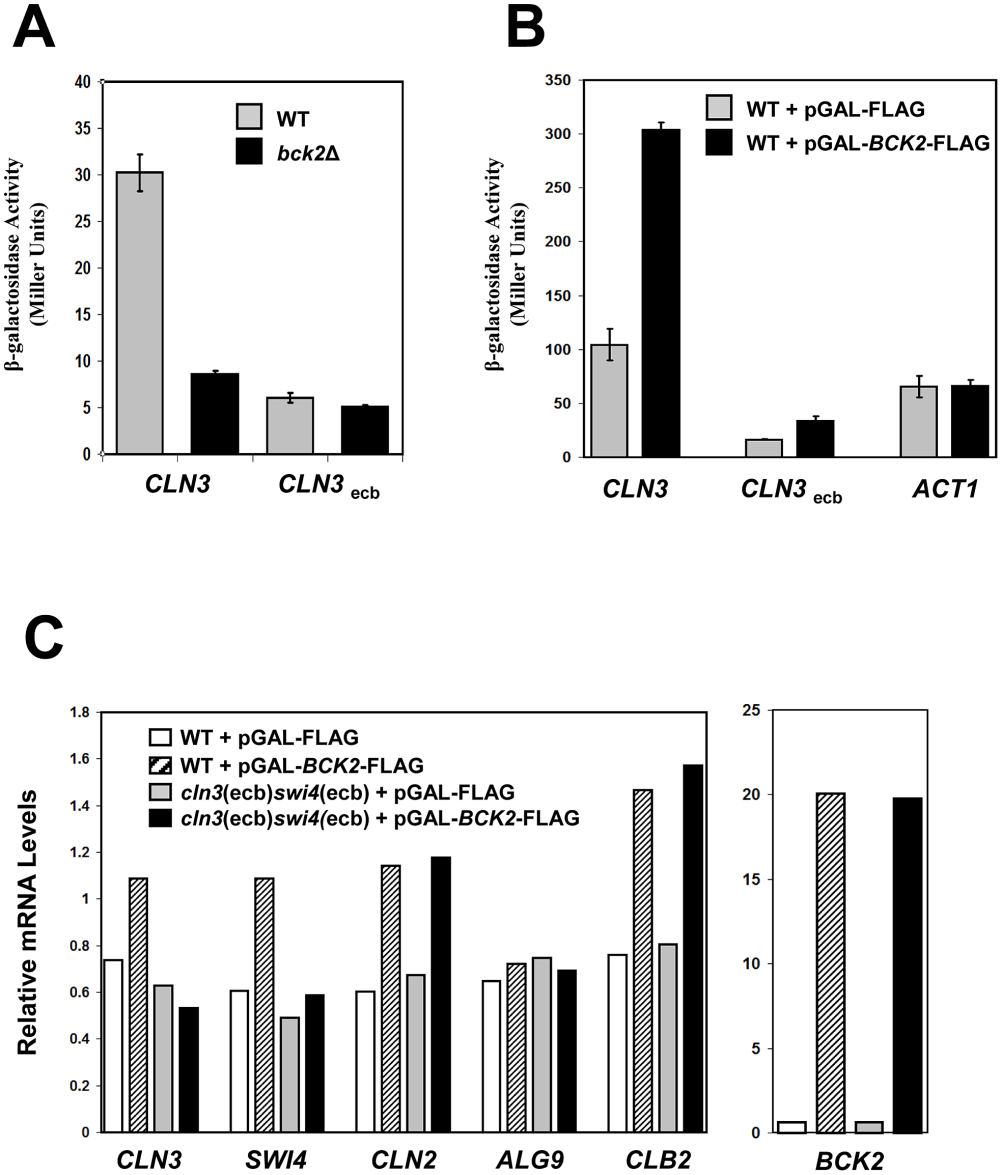
Bck2 requires intact ECB elements for transcriptional activation. (A) WT (grey bars) or *bck2*Δ (black bars) yeast transformants harboring a *CLN3*-*lacZ* or *CLN3*_ecb_-*lacZ* reporter plasmid were grown to mid-log phase in selective medium and subjected to quantitative β-galactosidase assays. (B) WT yeast strains containing a *CLN3*-*lacZ*, *CLN3*ecb-*lacZ* or *ACT1*-*lacZ* reporter plasmid were co-transformed with pGAL-*BCK2*-FLAG (black bars) or vector (grey bars) and grown to mid-log phase in selective medium containing galactose (inducing conditions) and subjected to quantitative β-galactosidase assays to measure *lacZ* expression. Y-axis values are expressed in Miller units. Error bars reflect values obtained from 3 independent transformants in separate experiments. (C) A WT (BY2125) strain and a strain lacking functional ECB elements in the *CLN3* and *SWI4* promoters (BY2680; *cln3*(ecb)*swi4*(ecb)) were transformed with pGAL-*BCK2*-FLAG (hatched bars in WT; black bars in mutant) or vector (white bars in WT; grey bars in mutant). Transformants were grown to saturation in plasmid selective medium and subcultured in YPGal to mid-log phase before harvesting for quantification of mRNA levels by Q-PCR analysis using *ACT1* mRNA levels as a normalizing control. Relative enrichment of *CLN3*, *SWI4*, *CLN2*, *ALG9* and *CLB2* mRNA normalized against *ACT1* mRNA is shown in the left panel. Relative enrichment of *BCK2* mRNA from the same samples normalized against *ACT1* mRNA is shown in the right panel.

The observation that Bck2 is required for proper transcription of M/G_1_ genes that contain Mcm1-binding sites suggested that Bck2 may function through ECB elements. To test this hypothesis, we assayed the effect of *BCK2* deletion on expression of *lacZ* reporter plasmids containing either a WT *CLN3* promoter that has intact ECB elements or a mutated *CLN3* promoter that lacks functional ECB elements [50]. Deletion of *BCK2* in combination with mutation of ECB elements caused a level of reporter gene expression similar to that seen with either perturbation alone (Figure 5A), indicating that Bck2 acts through ECB elements in order to control expression of M/G_1_ genes.

To gather more evidence that Bck2 works through ECB elements, we next tested the effects of *BCK2* overexpression on ECB-containing reporter gene expression. For this experiment, we used a *lacZ* reporter gene driven by a version of the *CLN3* promoter in which ECB elements were mutated. As previously seen [50], when ECB elements were mutated, expression of *CLN3pr*-*lacZ* in wild-type cells was substantially reduced (Figure 5B). Overexpression of *BCK2* induced expression of the wild-type *CLN3pr*-*lacZ* reporter gene approximately 3-fold, in a manner that was largely dependent on intact ECB elements (Figure 5B). We conclude that ECB elements are required for Bck2 to maximally activate transcription through the *CLN3* promoter. To substantiate the requirement of ECB elements in transcriptional activation of M/G_1_-regulated genes by overproduced Bck2, we next assayed the effects of *BCK2* overexpression on expression of the endogenous *CLN3* and *SWI4* genes. We compared the expression of *CLN3* and *SWI4* in a wild-type strain to a strain where ECB elements in the promoters of both *CLN3* and *SWI4* were mutated (*cln3*(ecb)*swi4*(ecb)) [49]. Consistent with our reporter gene assays, overproduction of Bck2 increased *CLN3* and *SWI4* transcript levels in a WT strain, but not the *cln3*(ecb)*swi4*(ecb) mutant strain (Figure 5C), indicating that Bck2 functions through ECB elements in endogenous M/G_1_ promoters.

### Bck2 Also Activates Expression of G_1_/S and G_2_/M Genes

As previously seen, overproduction of Bck2 also increased *CLN2* expression in WT cells [12], [51]; however, this induction was entirely independent of the ECB elements in the *CLN3* and *SWI4* gene promoters (Figure 5C). This result suggests that the induction of *CLN2* transcription by overexpressed *BCK2* is not an indirect consequence of increased *CLN3* and *SWI4* expression. Similarly, the induction of *CLB2* expression by overexpressed *BCK2* [12], [51] was also independent of the ECB elements in the *CLN3* and *SWI4* promoters (Figure 5C), again suggesting that the *CLB2* induction is not an indirect effect of defects in M/G_1_ phase gene expression. Finally, overexpressed *BCK2* did not alter expression of *ALG9*, a non-ECB containing gene, indicating that Bck2 is not an activator of global transcription. Together, our analyses of the effects of *BCK2* deletion and overexpression show that Bck2 activates *CLN3* and *SWI4* transcription in an ECB-dependent manner, while also promoting expression of G_1_/S (*CLN2*) and G_2_/M-regulated (*CLB2*) genes by a mechanism that does not depend on its effect on early G_1_ genes.

### Bck2 Localizes to M/G_1_, G_1_/S, and G_2_/M Phase Promoters

**Figure 6:**
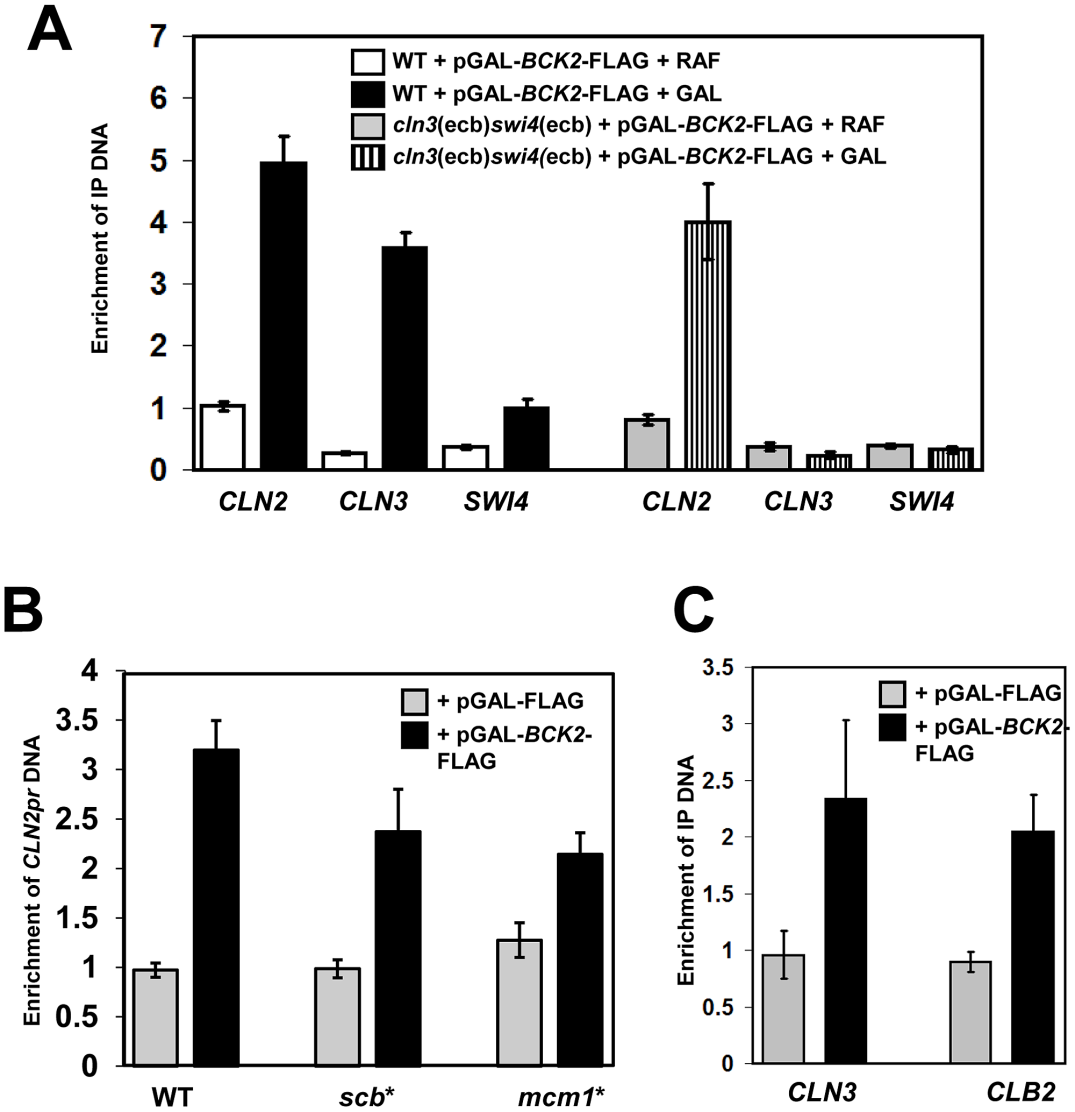
Bck2 localizes to the promoters of M/G_1_, G_1_/S and G_2_/M genes. (A) Bck2 localization to M/G_1_ genes depends on ECBs. WT strain (BY2125; W303) or a strain containing mutated ECB elements in the *CLN3* and *SWI4* promoters (BY2680; *cln3*(ecb)*swi4*(ecb)) was transformed with a pGAL-*BCK2*-FLAG plasmid and grown separately in raffinose- (non-inducing conditions) or galactose-containing medium (inducing conditions) to mid-log phase. Cultures were harvested and anti-FLAG ChIPs were analyzed for *CLN2*, *CLN3* and *SWI4* promoter DNA by Q-PCR. The Y-axis measures enrichment of promoter DNA for the target gene indicated relative to enrichment of non-promoter DNA from an untranscribed region of chromosome II. (B) Bck2 localization to the *CLN2* promoter is reduced when SCBs or Mcm1-binding sites are mutated. Vector and pGAL-BCK2-FLAG were transformed into WT (GC46), and strains with 3 SCBs mutated (yLB76-scb*) and 2 Mcm1-binding sites mutated (yLB76-mcm1*), cells were grown in inducing conditions, and Bck2-Flag localization to the *CLN2* promoter was analyzed by Q-PCR. (C) Bck2 localizes to the *CLB2* promoter. WT cells containing vector or pGAL-BCK2-FLAG were grown in inducing conditions and Bck2-Flag localization to the *CLN3* and *CLB2* promoter was analyzed by ChIP.

Since Bck2 functions through ECB elements (Figure 5) and physically interacts with Mcm1 (Figure 2), we next asked if Bck2 localized to the promoter regions of M/G_1_-phase genes. We first used chromatin immunoprecipitation (ChIP) with a strain carrying a TAP-tagged allele of Bck2 to assess association of Bck2 with various promoters. We detected a reproducible enrichment of promoter DNA in the Bck2 ChIP, but the signal was very low relative to Swi4 or Mcm1 ChIPs (data not shown). To improve our assay, we repeated the ChIP experiment using a strain in which a FLAG-tagged derivative of Bck2 was conditionally overproduced (Figure 6A). Under inducing conditions (galactose), Bck2-FLAG IPs were enriched in *CLN2*, *CLN3* and *SWI4* promoter DNA relative to non-inducing conditions (raffinose) (Figure 6A) or vector control (data not shown). The enhanced enrichment of *CLN3* promoter DNA compared to *SWI4* promoter DNA in Bck2 IPs likely reflects the presence of more ECB elements in the *CLN3* promoter (6 versus 1). Association of Bck2 with the *CLN3* and *SWI4* promoters was entirely dependent on the presence of ECB elements, while association with the *CLN2* promoter was unaffected, consistent with our gene expression analysis (Figure 5). Our findings are supported by a recent study that identified Bck2 as a constituent of DNA-bound complexes containing Mcm1 [52]. We conclude that Bck2 localizes to the promoters of *CLN3* and *SWI4* in a manner that depends on ECB elements.

Bck2 also localized to the *CLN2* promoter in our ChIP assays. The *CLN2* promoter contains three SCB elements, which are required for cell-cycle-specific gene expression, and two Mcm1 binding sites, which contribute to the presence of a nucleosome depleted region but have no effect on average expression levels or cell-cycle-specific expression [31]. We assayed the ability of Bck2 to localize to the *CLN2* promoter in strains that contained mutations in either set of elements [31]. Mutation of either the SCBs (scb*) or the Mcm1-binding sites (mcm1*) led to a modest reduction in Bck2 localization to the *CLN2* promoter (Figure 6B), suggesting that Bck2 may be recruited to *CLN2* by either SBF or Mcm1. The SBF-dependence of Bck2 recruitment to the *CLN2* promoter was further supported by a reduced Bck2 ChIP in the absence of *SWI4* or *SWI6* (Figure S2).

Since we saw that *BCK2* was required for proper expression of *CLB2* (Figure 4), and that overproduced Bck2 led to increased *CLB2* RNA levels (Figure 5C), we next asked whether Bck2 could also localize to the *CLB2* promoter. Mcm1 binds to the *CLB2* promoter in cooperation with another DNA-binding protein Fkh2, which is activated by Ndd1 [25]–[29]. Our ChIP experiments revealed that Bck2 localized to the *CLB2* promoter with an efficiency comparable to that seen with the *CLN3* promoter (Figure 6C), and the binding was partially dependent on *FKH2* (Figure S3).

### Bck2 May Compete with Yox1 for Access to Mcm1 on ECB Elements

**Figure 7:**
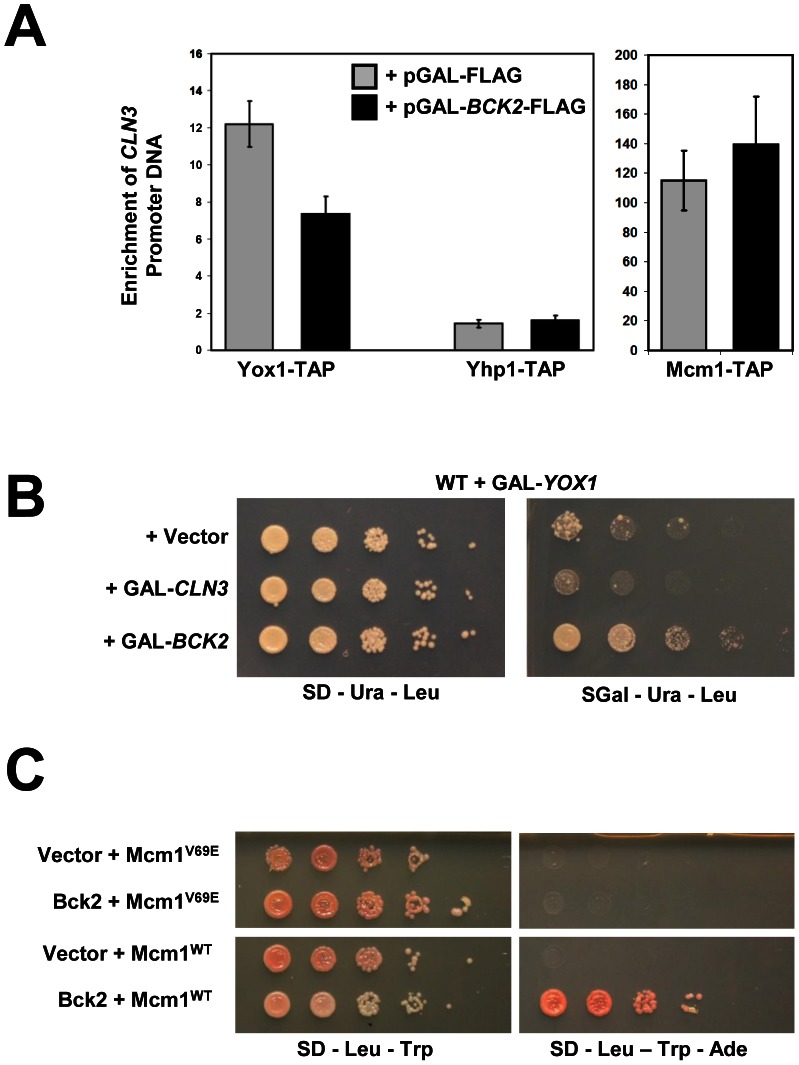
Bck2 may compete with Yox1 for interaction with Mcm1. (A) *YOX1*-TAP, *YHP1*-TAP and *MCM1*-TAP strains carrying a pGAL-*BCK2*-FLAG plasmid (black bars) or vector (grey bars), were individually grown in plasmid selective medium containing galactose (inducing conditions) to mid-log phase. Cultures were harvested and anti-TAP ChIPs were performed using IgG sepharose resin and analyzed for *CLN3* promoter DNA using Q-PCR. Error bars reflect values obtained after multiple Q-PCR runs from the same experiment. (B) A WT strain (Y7092) was co-transformed with a GAL-*YOX1* (*URA3*) plasmid and one of Vector, GAL-*CLN3*, or GAL-*BCK2* (*LEU2*) plasmid. Transformant cultures of equivalent optical density were spotted in serial 5-fold dilutions on selective medium that was either non-inducing (glucose) or inducing (galactose), and incubated for 72 h at 30°C. (C) A yeast two-hybrid bait strain (Y8930) carrying either plasmid *ADH1*-*GAL4* DB (vector) or plasmid *ADH1*-*GAL4* DB-*BCK2* Fragment 11 (Bck2) were mated to yeast two-hybrid prey strain (Y8800) carrying either *ADH1*-*GAL4* AD-*MCM1* ^WT^ or *ADH1*-*GAL4*-*MCM1* ^V69E^ to create diploid yeast strains. Diploids were spotted in serial 10-fold dilutions on double plasmid selection medium, or medium where growth is proportional to transcription of the *ADE2* gene, and incubated for 48–72 h at 30°C.

As noted earlier, the closely related homeodomain proteins Yox1 and Yhp1 act as repressors of Mcm1 by interacting directly with Mcm1 at DNA binding sites adjacent to the actual Mcm1 DNA binding site within the ECB [18]. Activation of Mcm1 on ECB elements correlates with removal of Yox1 from ECB elements, while deletion of *YHP1* has little effect on the level or periodicity of gene expression, and Yhp1 is not part of the predominant complex at ECB elements [18]. These observations suggested that Bck2 may activate *CLN3* and *SWI4* transcription through ECB elements by promoting the removal of Yox1. To test this hypothesis, we first assessed Yox1 binding to ECB elements within the *CLN3* promoter when Bck2 levels were elevated. Overexpression of *BCK2* significantly reduced the amount of Yox1 associated with the *CLN3* promoter (Figure 7A; left panel), implicating Bck2 in Yox1 removal. Yhp1 was not localized to the *CLN3* promoter to the same extent as Yox1, nor was the association affected by *BCK2* overproduction, consistent with a secondary role for Yhp1 [18]. Consistent with previous reports that Mcm1 remains localized to promoters throughout the cell cycle [18], we observed that Mcm1 localization was not significantly affected by *BCK2* dosage (Figure 7A, right panel). These experiments suggest that Bck2 overproduction may lead to the displacement of Yox1 repressor from the *CLN3* promoter, which is correlated with activation of M/G_1_-regulated genes [18].

Overexpression of *YOX1* is toxic, likely because high levels of Yox1 cause constitutive repression of its target genes [18]. To test the model that Bck2 may compete with Yox1 binding to Mcm1 at ECB elements, we asked if the toxicity caused by *YOX1* overexpression was suppressed by concurrent overexpression of *BCK2*. Previous work on the *CLB2* gene cluster, which is expressed at the G_2_/M phase transition, has shown that Mcm1 acts as a common scaffold for recruitment of Yox1 and the forkhead protein Fkh2 [30]. These physical interactions with Mcm1 are mutually exclusive and are mediated by distinct Yox1 and Fkh2 DNA binding elements that flank a central Mcm1 DNA binding site. When bound to Mcm1 on promoters, Fkh2 recruits the positively acting co-regulator Ndd1 in order to activate the *CLB2* cluster genes [26]. Constitutive overexpression of *YOX1* inhibits Ndd1 binding to the Yox1-regulated *SPO12* promoter [30], consistent with a mechanism based on mutual exclusivity between an activator and repressor. Our observation that overproduction of Bck2 leads to reduced Yox1 on the *CLN3* promoter (Figure 7A) suggested that the *YOX1* dose-lethality phenotype might be suppressed by concurrently overproducing *BCK2*. Indeed, we observed that overexpression of *BCK2* was able to significantly suppress the lethality caused by overexpressing *YOX1* (Figure 7B). Overexpression of *CLN3* failed to rescue the *YOX1* overexpression phenotype, suggesting that the *BCK2* rescue does not simply reflect an indirect effect of reduced G_1_ phase. Although the suppression of *YOX1* toxicity by overexpressed *BCK2* does not show that the effects are direct, it is consistent with a competitive relationship between Bck2 and Yox1 for interaction with the Mcm1 scaffold.

Mcm1 contains a hydrophobic pocket found on the surface of the MADS DNA-binding domain, and mutation of this pocket by the introduction of a V69E mutation disrupts interaction with Fkh2 [53]–[55], which prevents binding of both Yox1 and Fkh2 to Mcm1 [30]. We hypothesized that the competitive binding mechanism that allows Mcm1 to activate genes transcribed at the G_2_/M transition may also function for genes transcribed at the M/G_1_ transition. Specifically, we wondered whether Bck2 might activate Mcm1 at ECB elements through competition with Yox1 for binding to Mcm1. Consistent with our hypothesis, we found that the Y2H-based interaction between Bck2 and Mcm1 was abolished in the Mcm1^V69E^ mutant (Figure 7C). We conclude that Bck2 may act to remove Yox1 by a competitive binding mechanism similar to that seen at G_2_/M phase promoters [30].

## Discussion

### Bck2 Activates Early G_1_ Genes by Interacting with Mcm1 at ECB Elements

**Figure 8:**
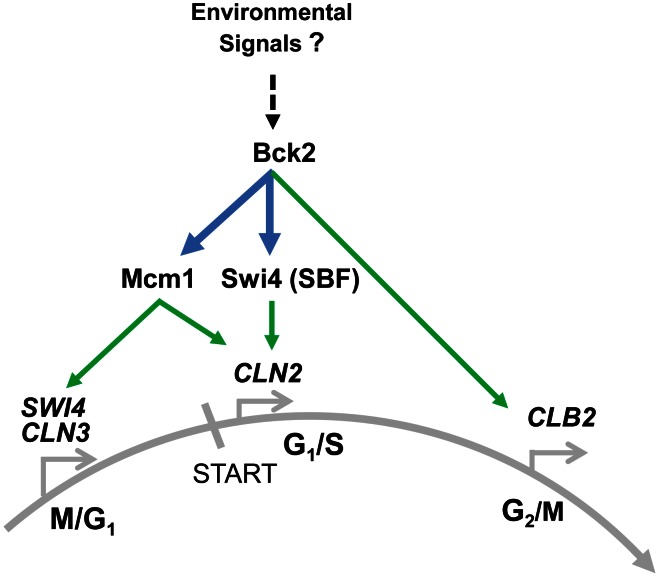
Summary of Bck2-dependent regulation of cell cycle gene expression. In pre-START cells Bck2 binds to Mcm1 and promotes expression of M/G_1_ genes such as *SWI4* and *CLN3*, which encode important constituents of the transcriptional ‘switch’ for START. Cln3 then contributes to activation of Swi4, a component of SBF, which induces expression of G_1_/S genes such as *CLN2*. Bck2 also induces *CLN2* expression directly through physical association with Swi4 and with Mcm1. In G_2_/M Bck2 promotes expression of *CLB2*, possibly through interaction with Mcm1. We speculate that Bck2 may be responding to environmental signals such as nutrients in its role of activating cell cycle gene expression (see text for details). Bold lines indicate interactions, both protein-protein (in blue) and protein-DNA (in green) described in this work.

Our work identifies a previously unappreciated role for Bck2 in the regulation of M/G_1_-specific transcription in budding yeast, and also reveals functions for Bck2 in regulating late G_1_ and G_2_/M genes (Figure 8). In contrast to the *CLB2* gene cluster, no positively acting partner protein had yet been found that cooperates with Mcm1 to regulate M/G_1_-expressed genes. We describe several observations showing that Bck2 functions to control Mcm1 activity on promoter elements to ensure the proper regulation of early G_1_ events. First, Bck2 is required to activate expression of the M/G_1_ genes *SWI4*, *CLN3*, *CDC6*, and *CDC47*. Second, we show a requirement for intact Mcm1-binding ECB elements within the promoters of *CLN3* and *SWI4*, consistent with the physical interaction between Bck2 and Mcm1 that we see by Y2H. Third, Bck2 localizes to early G_1_ promoters in an ECB-dependent manner.

Our finding that Bck2 is needed for expression of Mcm1-regulated genes in M/G_1_ phase may explain previous observations that Bck2 activates G_1_/S gene expression in a pathway distinct from that involving Cln-Cdc28 activation of promoter-bound SBF/MBF. For example, high-copy *BCK2* activates SBF/MBF target genes in a *cdc28*-*4* mutant [13] and suppresses the G_1_-arrest of a *cln1*Δ*cln2*Δ*cln3*Δ strain [11]. These activities likely reflect increased *SWI4* expression, because Bck2 activates *SWI4* transcription [12], [48], and high-copy suppression by *BCK2* of the G_1_-arrest of a *cln1*Δ*cln2*Δ*cln3*Δ strain requires *SWI4* [11]. Importantly, *SWI4* is induced in a *cdc28*-*13* mutant [7]. There is no evidence that Cdk activity is required for activation of M/G_1_-expressed genes.

### Bck2-Dependent Activation of G_1_/S Genes

Bck2 has also been shown to activate G_1_/S genes, in a manner that is both partially dependent and partially independent of SBF/MBF activity. For example, overproduction of Bck2 stimulates expression of an *SCB-lacZ* reporter gene, activation of which is strictly dependent on SBF [12]. Large-scale mass spectrometry experiments reveal an interaction between Bck2 and Swi4 [56] and we see a weak Y2H interaction between *BCK2* and *SWI4* (Figure 2). In our experiments, localization of Bck2 to the *CLN2* promoter was modestly reduced when the SCB sites were mutated (Figure 6B) and Bck2 localization was partially dependent on *SWI4* and *SWI6* (Figure S2), suggesting that SBF has a role in recruiting Bck2. However, consistent with an SBF-independent activity, Bck2 can activate several natural SBF/MBF target gene promoters in the absence of either *SWI4* or *MBP1* [13], or the elements SBF/MBF bind [12]. In our experiments, mutation of the Mcm1-binding sites in the *CLN2* promoter also modestly reduced Bck2 binding (Figure 6B), suggesting that Mcm1 also contributes to Bck2 recruitment to the *CLN2* promoter. Since SCB elements are much more important for proper *CLN2* expression than Mcm1-binding sites [31], it is likely that SBF has a more important role than Mcm1 in recruiting Bck2 to the *CLN2* promoter.

### Bck2-Dependent Activation of G2/M Genes

We present several lines of evidence that suggest a role for Bck2 in activation of G_2_/M genes. First, a *bck2*Δ strain has delayed *CLB2* expression (Figure 4). Second,overexpression of *BCK2* leads to increased expression of *CLB2* [12] (Figure 5C), and other G_2_/M genes [51]. Third, we show that Bck2 localizes to the *CLB2* promoter (Figure 6C), in a manner dependent on *FKH2* (Figure S3). Activation of G_2_/M genes is controlled by Ndd1, which is recruited to promoters by binding Fkh2 [25]–[29]; it is not clear why *FKH2* is involved in Bck2 localization to the *CLB2* promoter. One possibility is that Bck2 binds to both Fkh2 and Mcm1 at G_2_ promoters, similar to its binding to SBF and Mcm1 at G_1_/S promoters; however, we did not detect an interaction between *BCK2* and *FKH2* in a pairwise Y2H assay (data not shown). Regardless, the mechanism of action of Bck2 at G_2_/M promoters must differ from that at M/G_1_ promoters where Bck2 appears to act on Mcm1 alone. Unlike the G_2_/M promoters, M/G_1_ promoters [6] do not contain DNA binding sites for Fkh2 [29] or other positive regulators, indicating that induction of M/G_1_ [18] genes depends on a positively-acting protein that presumably acts through Mcm1.

### Bck2 as an Activator of Other Mcm1-Regulated Genes

Mcm1, together with class-specific regulators, controls the expression of several different groups of genes in addition to the cell-cycle-regulated genes discussed above: (1) activation of genes involved in mating together with Ste12; (2) regulation of cell-type-specific genes together with α1 or α2; and (3) control of genes involved in arginine metabolism together with Arg80 (for review see [57]). Other observations suggest that Bck2 may be involved in regulating expression of other classes of Mcm1-dependent genes. First, *BCK2* was identified as a gene whose overexpression strongly induced the expression of *FUS1* reporter genes [58]. Second, a more recent study examining transcriptional profiles found that overexpression of *BCK2* led to expression of cell-cycle-regulated genes from multiple cell-cycle stages, consistent with our findings [51]. Furthermore, overexpression of *BCK2* induced a large number of genes involved in mating, but not genes involved in cell type or nitrogen metabolism [51]. Indeed these authors suggested that Bck2 may elicit gene expression via Ste12-Mcm1. We have shown that Bck2 acts through Mcm1 to promote expression of three classes of cell cycle genes; we suggest that Bck2 likely regulates mating genes in a similar manner.

### Regulation of Bck2

We have shown in this work that Bck2 regulates cell cycle gene expression. We suggest that the role of Bck2 may be to fine-tune expression of different classes of Mcm1-regulated genes. How might Bck2 itself be regulated? Bck2 is rich in serine and threonine residues and has been shown to be phosphorylated at multiple sites at different stages of the cell cycle [59], [60]. Bck2 has been linked to several cell-cycle-regulating kinases and phosphatases in genetic studies (the Protein Kinase C pathway [16], [48], Sit4 [12], [61], Cdc28 [13], [48], Cbk1 [16]) and in mass spectrometry and phosphopeptide analyses (Cdc15 [62], Cdc28 [60], Fus3 and Kss1 [56]). Thus Bck2 may play a role in linking detection of nutrients or other conditions that affect cell cycle progression to Mcm1-dependent gene expression (Figure 8). For example, during early G_1_ phase, yeast cells assess nutrient status, in part through the Tor pathway and the phosphatase Sit4 [63]. *BCK2* has been proposed to function in the *SIT4* pathway of *CLN* activation [12], [61]. Both Tor and Sit4 signaling are also required for proper M/G_1_ gene expression [63], [64], and like *tor1*Δ cells and *sit4*Δ cells, *bck2*Δ cells are rapamycin sensitive [16], [63], [65]. Thus Bck2 may be part of the signal transduction pathway linking nutrient status to passage through M/G_1_ of the cell cycle. Consistent with a potential role for Bck2 in integrating environmental and cell cycle signals, our Y2H screen identified several transcription factors and regulators with established functions in regulating gene expression in response to various nutrients or stresses, including nutrient sensing through the TOR pathway (see Results and Figure 2). How Bck2 may work together with various proteins to regulate nutrient and stress responses remains to be determined.

### Bck2 Mode of Action

Based on the high level of similarity between budding yeast Mcm1 and human Serum Response Factor (SRF) [30], [54], [55], [66]–[68], the Bck2 protein might represent a budding yeast analog of a specific SRF co-activator in mammalian cells. For example, the myocardin/MKL family members are SRF co-activators which are enriched at target promoters in serum-rich medium [69]. Our discovery of Bck2 as a cofactor for Mcm1, coupled with accumulating evidence for a role for Bck2 in nutrient sensing, suggests that Bck2 binding may also increase under nutrient-rich conditions. Further studies will be required to firmly test the analogy between the Mcm1-Bck2 and SRF-MKL pathways and to illuminate how Bck2 may link gene expression, the cell cycle machinery and environmental signals to ensure appropriate cell proliferation.

## Materials and Methods

### Yeast Strains and Plasmids

Yeast strains used in this study (Table 1) were derivatives of either S288C or W303, with the exception of the Y2H strains. Plasmids are described in Table 2. Yeast cultures were grown in YEP (1% yeast extract, 2% bactopeptone) supplemented with 2% glucose. Synthetic minimal medium supplemented with the appropriate nutrients was used to select for plasmid maintenance and gene replacements. Yeast transformation and general manipulation of yeast cells were performed using standard techniques.

**Table 1:** *Saccharomyces cerevisiae* strains used in this study.

Strain	BY#	Genotype	Source
BY4741	1623	*MAT* **a** *his3*Δ*1 leu2*Δ*0 met15*Δ*0 ura3*Δ*0*	[74]
BY4742	1624	*MAT*α *his3*Δ*1 leu2*Δ*0 lys2*Δ*0 ura3*Δ*0*	[74]
BY3015	3015	*MAT* **a** *cln3*Δ::Hph *bck2*Δ::Nat *his3*Δ*1 leu2*Δ*0 met15*Δ*0 ura3*Δ*0 lys2*Δ*0* pGAL-*CLN3*-*URA3*	This study
Y8930	4889	*MAT*α *trp1-901 leu2-3,112 his3-200 ura3-52 gal4Δ gal80Δ LYS2::GAL1-HIS3 GAL2-ADE2 met2::GAL7-lacZ cyh2*	[72]
Y8800	4890	*MAT* **a** *trp1-901 leu2-3,112 his3-200 ura3-52 gal4Δ gal80Δ LYS2::GAL1-HIS3 GAL2-ADE2 met2::GAL7-lacZ cyh2*	[72]
Y8890	4896	*MAT* **a** *cdc20*-*3::Kan his3*Δ*1 leu2*Δ*0 met15*Δ*0 ura3*Δ*0*	[75]
	4897	*MAT*α *cdc20*-*3::Kan bck2*Δ::Nat *his3*Δ*1 leu2*Δ*0 met15Δ0 ura3Δ0*	This study
BY2125	4898	*MAT* **a** *ade2*-*1 his3*-*11*, *15 leu2*-*3*, *112 trp1*-*1 ura3 can1*-*100 ssd1*-*d*	[49]
BY2680	4899	*MAT* **a** *ade2*-*1 his3*-*11*, *15 leu2*-*3*, *112 trp1*-*1 ura3 can1*-*100 ssd1*-*d cln3ecb5 swi4ecb*	[49]
*YOX1*-TAP	4900	*MAT* **a** *his3*Δ*1 leu2*Δ*0 met15*Δ*0 ura3*Δ*0 YOX1- TAP::HIS3*	[32]
*YHP1*-TAP	4901	*MAT* **a** *his3*Δ*1 leu2*Δ*0 met15*Δ*0 ura3*Δ*0 YHP1-TAP::HIS3*	[32]
*MCM1*-TAP	4902	*MAT* **a** *his3*Δ*1 leu2*Δ*0 met15*Δ*0 ura3*Δ*0 MCM1-TAP::HIS3*	[32]
Y7092	4903	*MAT*α *his3*Δ*1 leu2*Δ*0 met15*Δ*0 ura3*Δ*0 can1*Δ*::STE2pr-HIS3 lyp1*Δ	[76]
Y1335	4904	*MAT*α *bck2*Δ::Nat *his3*Δ*1 leu2*Δ*0 met15*Δ*0 ura3*Δ*0 can1*Δ::*STE2pr*-*HIS3 lyp1*Δ	[76]
	4905	*yox1*Δ::*kan yhp1*Δ::*LEU2 his3*Δ*1 leu2*Δ*0 met15Δ0 ura3Δ0*	This study
GC46	4980	*MAT* **a** *MYO1::MYO1-mCherry-SpHIS5 ADE2*	[31]
yLB76-mcm1*	4977	*MAT* **a** CLN2:: Cln2pr-mcm1*-GFP-Cln2pest-CaURA3 MYO1::MYO1-mCherry-SpHIS5 ADE2	[31]
yLB76-scb*	4979	*MAT* **a** CLN2:: Cln2pr-scb*-GFP-Cln2pest-CaURA3 MYO1::MYO1-mCherry-SpHIS5 ADE2	[31]

Strains are isogenic with BY4741, except for BY2125 and BY2680, and GC46, yLB76-mcm1*, and yLB76-scb*, which are isogenic to W303, and the Y2H strains Y8930 and Y8800. Where strains are not listed in this table, strains from the yeast gene-deletion set [77] were used.

**Table 2:** Plasmids used in this study.

Plasmid	BA#	Description	Source
pDONR201	346V	Gateway donor vector	Invitrogen
pDEST DB	2415	*LEU2 CEN ADH1*_pr_-GAL4 DB	[72]
pDESTDB-GAL4-*BCK2*-1	2416	*BCK2* aa1-851 (Fragments 1–20)	This study
pCLM771	2436	4xP-*lacZ URA3* 2μ	[47]
pBD1790	2437	*CLN3*-*lacZ URA3*	[50]
pBD1867	2438	*CLN3*(ecb)-*lacZ URA3*	[50]
pBD1577	2439	*SWI4*-*lacZ URA3*	[50]
pBD1637	2440	*CDC6*-*lacZ URA3*	[50]
pBD1951	2441	*CDC47*-*lacZ URA3*	[50]
*ACT1*-*lacZ*	2442	*ACT1*-*lacZ URA3*	[78]
pGAL-FLAG	350V	*LEU2 CEN GAL1*_pr_-*FLAG*	[79]
pGAL-*BCK2*-FLAG	2412	*LEU2 CEN GAL1*_pr_-*BCK2-FLAG*	[79]
pGAL-*CLN3*-FLAG	2443	*LEU2 CEN GAL1*_pr_-*CLN3-FLAG*	[79]
pGAL-*YOX1*	2444	*URA3 CEN GAL1pr-YOX1*	[80]
pDEST32	401V	*LEU*2 *CEN ADH1*_pr_-*GAL4* DB	Invitrogen
pDEST32-*BCK2*	2414	*LEU*2 *CEN ADH1*_pr_-*GAL4 DB-BCK2*	This study
pDEST22-*MCM1* ^WT^	2445	*TRP1 CEN ADH1*_pr_-*GAL4* AD-*MCM1*	This study
pDEST22-*MCM1* ^V69E^	2446	*TRP1 CEN ADH1*_pr_-*GAL4* AD-*MCM1* ^V69E^	This study

### Cloning and Construction of *BCK2* Truncations

Fragments of the *BCK2* gene were amplified from genomic DNA using PCR primers designed to be compatible with the Gateway system of recombinational cloning (Table S1). All PCR products were recombined into the donor vector pDONR201 using the BP clonase II system (Invitrogen) and positive clones were fully sequence-confirmed. *BCK2* truncations within pDONR201 were recombined into the destination vector pDEST-DB using the LR clonase II system.

### β-Galactosidase Assays

Quantitative β-galactosidase assays were performed as follows. Exponentially growing cells at an optical density at 600 nm of 0.2 to 0.25 were harvested. Extracts were prepared by vortexing the cells in 1 ml Z-buffer (0.1 M NaPO_4_ [pH 7.0], 0.01 M KCl, 1 mM MgSO_4_, 4 mM 2-mercaptoethanol)+20 µl 0.1% SDS+40 µl chloroform for 45 seconds. After 5 min, 0.2 ml of *o*-nitrophenylgalactoside (Sigma; at 4 mg/ml in Z buffer) solution was added and the reaction was incubated at 30°C until a slight yellowing was observed. The reactions were stopped by the addition of 0.5 ml of 1 M Na_2_CO_3_, and the samples were centrifuged for 3 minutes at 13,000× g. The A_420_ of the supernatant was determined. β-Galactosidase units were calculated using the following formula: units = (A_420_)*(1000)/(time of reaction, in minutes)*(volume of extract in assay = 1 ml)*(cellular concentration in OD600 values). For each time point, the assays were performed on three separate cultures, and the average is reported. Qualitative β-galactosidase overlay assays were performed as described [70].

### Complementation Analysis

Transformants of a *cln3*Δ*bck2*Δ p*GAL*-*CLN3* -*URA3* strain (BY3015) bearing *ADH1*-GAL4 DBD-*BCK2-LEU2* (1–20) plasmids were grown in plasmid selective medium, prepared at equivalent optical density and spotted in serial 10-fold dilutions onto plasmid selective medium containing either galactose lacking 5′FOA or glucose+5′FOA, and incubated for 48 h at 30°C.

### Genome-Wide Y2H Screen

The Y2H ORFeome method [71] was used to screen for Bck2-interacting proteins by mating a prey strain with a bait strain. The AD ORFeome (prey strain) is a pooled collection of AD-ORF plasmids [72] in a 96-well format. The pooled AD ORFeome [72] was grown in a 96-well culture block containing 600 µl per well of SD – Trp media at 30°C for 48 hours. Five µl of AD ORFeome culture were spotted from the 96-well culture block onto large YPD plates and allowed to dry. Next, 5 µl of a DBD ORF strain culture (bait strain transformed with a single DBD-ORF plasmid) was dispensed directly onto the ORFeome strain spots. Five diploid control strains were spotted onto the same plate at an empty location in order to ensure the quality of selection plates and to help evaluate the phenotype of interactions. The plate was incubated at 30°C until growth of spots was apparent before replica plating onto SD – Leu – Trp and grown for 3 days at 30°C in order to select only diploid yeast. This plate was replica plated onto a final selection plate containing SD – Leu – Trp – Ade and grown for 5 days until foci were observed. At least 3 foci per spot were picked, and subjected to colony PCR using primers homologous to the sequences flanking the ORF within the AD plasmid. Yeast cells were scraped from plates into 30 µl of lysis solution (2.5 mg zymolyase in 1 ml 1 M sorbitol), and incubated at 37°C for 15 minutes then 95°C for 5 minutes before addition of 120 µl of ddH_2_O. PCR reactions were performed in 25 µl volumes containing 5 µl of the yeast cell preparation described. PCR reactions were performed using 5 minute extension times in order to ensure that large ORF inserts were isolated. PCR reactions were electrophoresed on agarose gels, purified using the PureLink kit (Invitrogen) and sent for sequencing analysis.

### Direct Pair-Wise Y2H Assays

Yeast transformants carrying *ADH1*-*GAL4* DBD (vector; *LEU2*) or *ADH1*-*GAL4* DBD-*BCK2* Fragment 11 (Bck2) in a two-hybrid bait strain (Y8930) were mated to yeast transformants of a two-hybrid prey strain (Y8800) bearing specific *ADH1*-*GAL4-AD-ORF-TRP* plasmids. Diploids were selected by streaking cells onto double plasmid selection medium (SD – Leu – Trp). Diploids of equivalent optical density were spotted in serial 10-fold dilutions on double plasmid selection medium (SD – Leu – Trp), or medium where growth was proportional to transcription of the *ADE2* gene (SD – Leu – Trp - Ade), and incubated for 48 h at 30°C. Six diploid strains carrying different combinations of AD and DBD ORF-fusions [72], [73] were used as a spectrum of positive and negative controls.

### Cell Synchronization

A *cdc20*-*3* temperature-sensitive strain (BY4896, Table 1) was grown in YPD medium at 21°C, arrested in M phase by incubation at 37°C for 3.5 hours, and released into the cell cycle by transferring the culture back to 21°C. Arrest was determined by visualization of large budded cells under a light microscope.

### mRNA Purification and RT–qPCR

Total RNA was isolated by phenol-chloroform extraction and further purified using the RNeasy kit (Qiagen). RNA was transcribed into cDNA using the Superscript II Reverse Transcriptase kit (Invitrogen) and RNA was then removed by addition of NaOH. Reactions were run on the ABI 7500 system (Applied Biosystems) using standard Q-PCR conditions. Data were analyzed using ABI7500 system software. VIC and FAM labelled fluorogenic primers (ABI), used to detect *CLN2*, *ALG9*, *CLN3*, *SWI4*, *BCK2*, *CLB2* and *ACT1* cDNA, are described in Table S1.

### Mutagenesis

To construct the Mcm1^V69E^ yeast two-hybrid prey plasmid, the Mcm1^WT^ prey plasmid was subject to in vitro mutagenesis using the QuikChange site-directed mutagenesis kit (Stratagene).

### ChIP Assays

Yeast strains transformed with pGAL-FLAG plasmids were grown in raffinose-containing minimal media overnight, and then grown separately in raffinose- (non-inducing conditions) or galactose-containing medium (inducing conditions) to mid-log phase. Cultures were harvested and anti-FLAG ChIPs were analyzed for *CLN2*, *CLN3*, *CLB2* and *SWI4* promoter DNA by TaqMan Q-PCR (Applied Biosystems) using primers with homology to target gene promoter DNA (Table S1). Enrichment of promoter DNA was determined relative to non-promoter DNA from an untranscribed region of chromosome II (Table S1).

## Supporting Information

Figure S1 Assays with *BCK2* truncations fused to the *GAL4* DNA binding domain. (A) Transcriptional activation by *BCK2* truncations in *lacZ* reporter assays. Yeast-two-hybrid bait strains, in which *GAL4* UAS elements drive expression of *ADE2*, *HIS3*, and *lacZ* reporter genes (Y8930), bearing truncated versions of the *BCK2* gene, *ADH1*-*GAL4* DB-*BCK2* (F1-20) (*LEU2*) plasmids were spotted onto plasmid selection medium, incubated for 48 h at 30°C, overlaid with a top agar solution containing X-Gal, and incubated at 30°C until blue color was seen (inset). To quantify the differences in transcriptional activity observed by the overlay assay, three independent isolates from a single transformation reaction were grown to mid-log phase in plasmid selective medium and subjected to quantitative β-galactosidase liquid assays to measure *lacZ* expression. Values are expressed in Miller Units. (B) Transcriptional activation by *BCK2* truncations in *ADE2* reporter assays. A yeast-two-hybrid bait strain where *GAL4* UAS elements drive expression of *ADE2*, *HIS3*, and *lacZ* reporter genes (Y8930) bearing *ADH1*-*GAL4* DB-*BCK2* (1–20) (*LEU2*) plasmids were spotted in serial 10-fold dilutions on plasmid-selection medium or medium where growth is proportional to transcription of the *ADE2* gene. Plates were incubated for 48 h at 30°C. (C) Complementation of a *cln3*Δ*bck2*Δp*GAL*-*CLN3* strain growth defect by *BCK2* truncation derivatives. Transformants of a *cln3*Δ*bck2*Δ *GAL*-*CLN3* (*URA3*) strain (BY3015) bearing *ADH1*-*GAL4* DBD-*BCK2* (1–20) plasmids (*LEU2*) were grown in plasmid selective medium, grown to equivalent optical density and spotted in serial 10-fold dilutions onto plasmid selective medium containing either galactose without 5-FOA or glucose+5-FOA. Plates were incubated for 48 h at 30°C.
(PDF)

Figure S2 Localization of Bck2-Flag and Swi4 to the *CLN2* promoter. WT (BY4741) and *swi4*Δ, *swi6*Δ and *mbp1*Δ strains from the deletion set containing pGAL-BCK2-FLAG were grown in inducing conditions and Bck2-FLAG (left panel) and Swi4 (right panel) recruitment to the *CLN2* promoter were assayed by ChIP.
(PDF)

Figure S3 Localization of Bck2-FLAG to the *CLB2* promoter. WT (BY4741) and a *fkh2*Δ strain from the deletion set containing vector or pGAL-BCK2-FLAG were grown in inducing conditions and Bck2-FLAG recruitment to the *CLB2* promoter were assayed by ChIP.
(PDF)

Table S1 Oligonucleotides used in this study. Y2H AD PCR Primers and *BCK2* Truncation Primers were used in constructing plasmids (underlined regions indicate *BCK2* sequence). Y2H AD Sequencing Primers were used to confirm that the sequence was correct. Q-PCR analysis of RNA and chromatin ChIP DNA was done using TaqMan with the primers and fluorogenic probes shown. The *mcm1-V69E* mutant was constructed with the mutagenic primers shown.
(XLS)
