# Supplementary figures and images for: Bck2 Acts through the MADS Box Protein Mcm1 to Activate Cell-Cycle-Regulated Genes in Budding Yeast
Source: PLoS Genet. 2013 May 9;9(5):e1003507. doi: 10.1371/journal.pgen.1003507 (PMC3649975; doi:10.1371/journal.pgen.1003507)

S1A

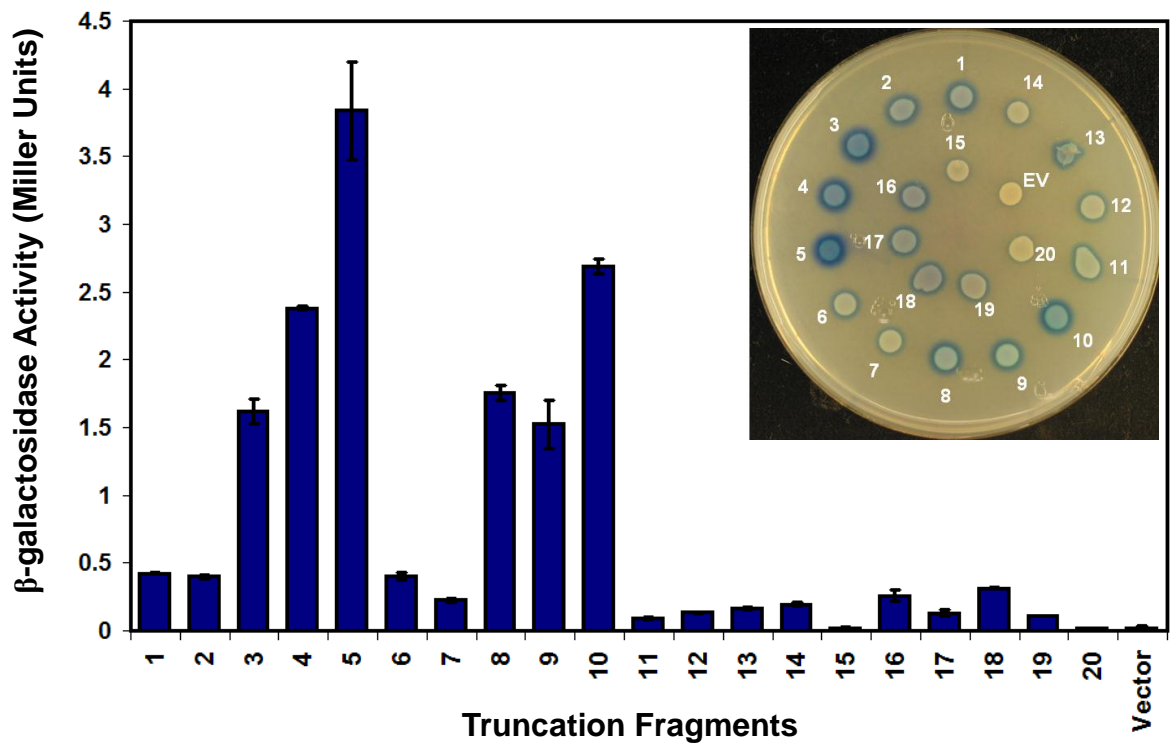

S1B

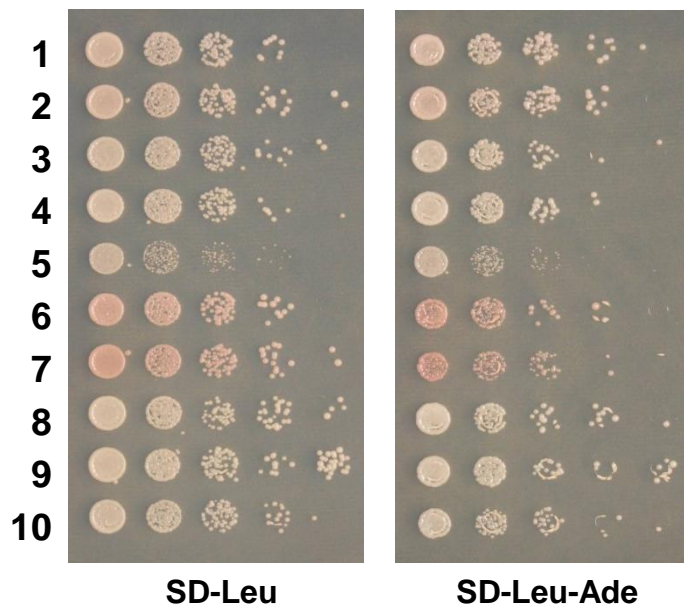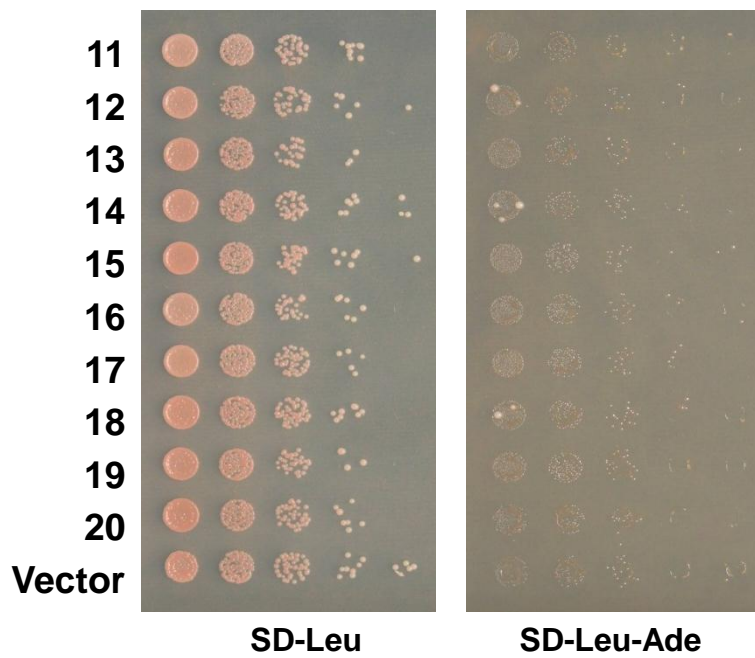

S1C

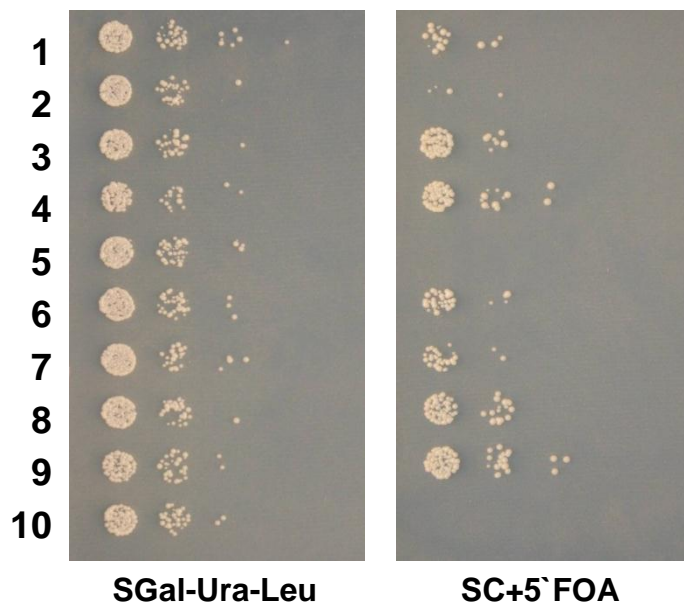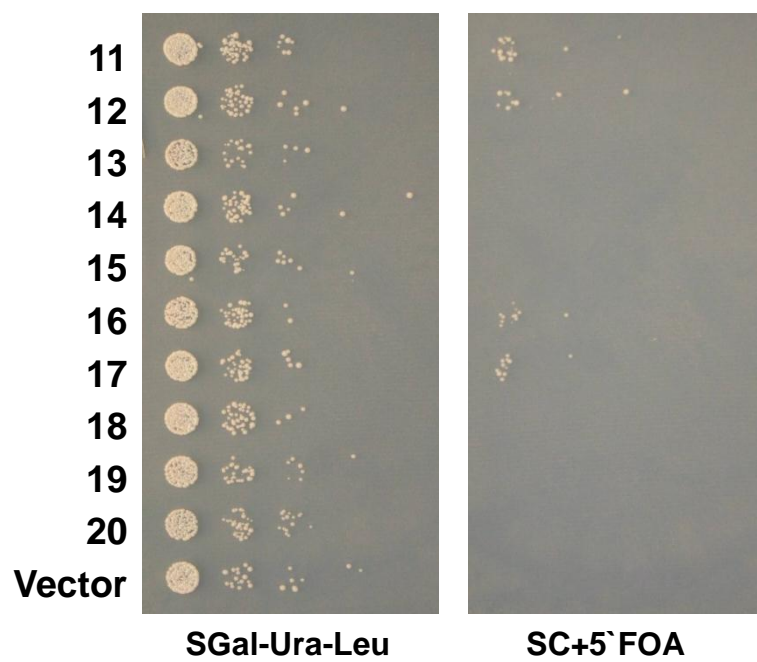

Supplement: Figure S1 — Assays with BCK2 truncations fused to the GAL4 DNA binding domain. (A) Transcriptional activation by BCK2 truncations in lacZ reporter assays. Yeast-two-hybrid bait strains, in which GAL4 UAS elements drive expression of ADE2, HIS3, and lacZ reporter genes (Y8930), bearing truncated versions of the BCK2 gene, ADH1-GAL4 DB-BCK2 (F1-20) (LEU2) plasmids were spotted onto plasmid selection medium, incubated for 48 h at 30°C, overlaid with a top agar solution containing X-Gal, and incubated at 30°C until blue color was seen (inset). To quantify the differences in transcriptional activity observed by the overlay assay, three independent isolates from a single transformation reaction were grown to mid-log phase in plasmid selective medium and subjected to quantitative β-galactosidase liquid assays to measure lacZ expression. Values are expressed in Miller Units. (B) Transcriptional activation by BCK2 truncations in ADE2 reporter assays. A yeast-two-hybrid bait strain where GAL4 UAS elements drive expression of ADE2, HIS3, and lacZ reporter genes (Y8930) bearing ADH1-GAL4 DB-BCK2 (1–20) (LEU2) plasmids were spotted in serial 10-fold dilutions on plasmid-selection medium or medium where growth is proportional to transcription of the ADE2 gene. Plates were incubated for 48 h at 30°C. (C) Complementation of a cln3Δbck2ΔpGAL-CLN3 strain growth defect by BCK2 truncation derivatives. Transformants of a cln3Δbck2Δ GAL-CLN3 (URA3) strain (BY3015) bearing ADH1-GAL4 DBD-BCK2 (1–20) plasmids (LEU2) were grown in plasmid selective medium, grown to equivalent optical density and spotted in serial 10-fold dilutions onto plasmid selective medium containing either galactose without 5-FOA or glucose+5-FOA. Plates were incubated for 48 h at 30°C. (PDF) [file pgen.1003507.s001.pdf]

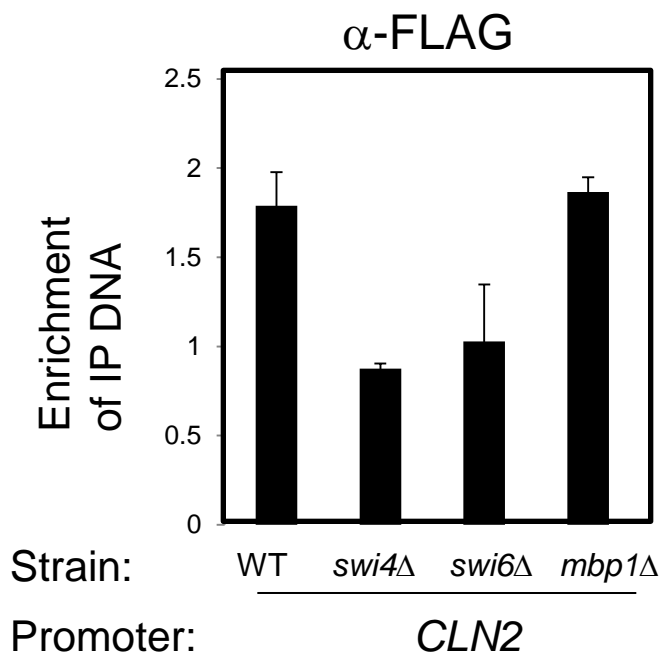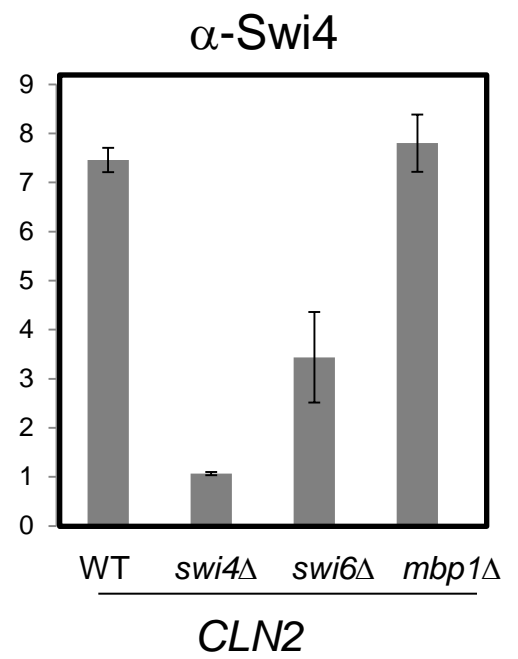

Supplement: Figure S2 — Localization of Bck2-Flag and Swi4 to the CLN2 promoter. WT (BY4741) and swi4Δ, swi6Δ and mbp1Δ strains from the deletion set containing pGAL-BCK2-FLAG were grown in inducing conditions and Bck2-FLAG (left panel) and Swi4 (right panel) recruitment to the CLN2 promoter were assayed by ChIP. (PDF) [file pgen.1003507.s002.pdf]

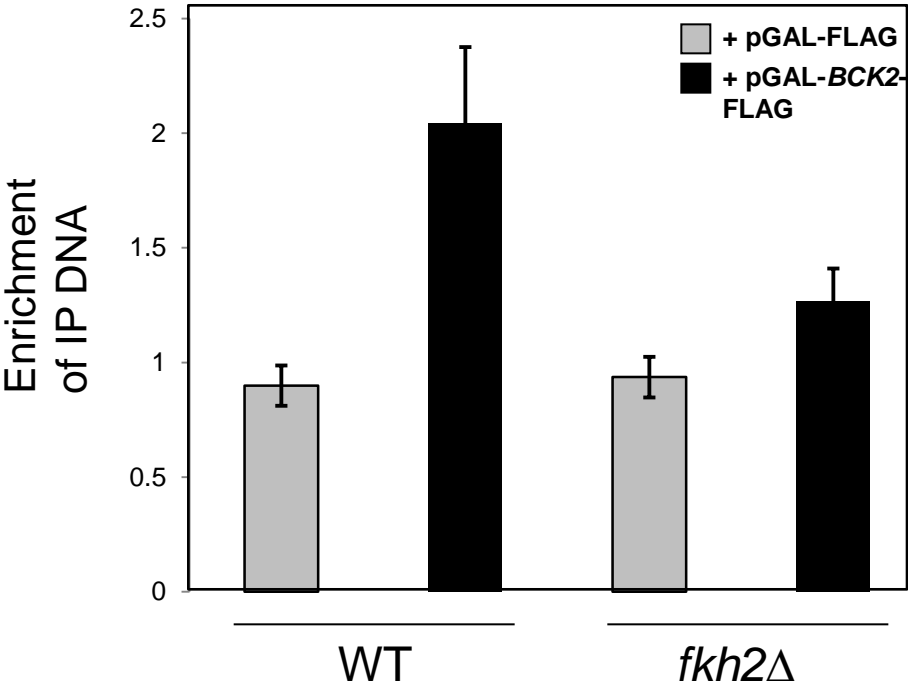

Supplement: Figure S3 — Localization of Bck2-FLAG to the CLB2 promoter. WT (BY4741) and a fkh2Δ strain from the deletion set containing vector or pGAL-BCK2-FLAG were grown in inducing conditions and Bck2-FLAG recruitment to the CLB2 promoter were assayed by ChIP. (PDF) [file pgen.1003507.s003.pdf]
